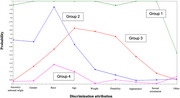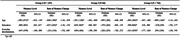# Latent Classes of Attributions for Everyday Discrimination and Associations with Episodic Memory Trajectories Among U.S. Black Women

**DOI:** 10.1002/alz70860_100597

**Published:** 2025-12-23

**Authors:** Tanisha G Hill‐Jarrett, Kendra D Sims, Ruijia Chen, Sydney Moseley, Justina F. Avila, Ketlyne Sol, Paris AJ Adkins‐Jackson, Kacie D Deters, M. Maria Glymour

**Affiliations:** ^1^ University of California, San Francisco, San Francisco, CA, USA; ^2^ Boston University, Boston, MA, USA; ^3^ Columbia University Irving Medical Center, New York, NY, USA; ^4^ University of Michigan, Ann Arbor, MI, USA; ^5^ Columbia University Mailman School of Public Health, New York, NY, USA; ^6^ University of California, Los Angeles Integrative Biology and Physiology (IBP), Los Angeles, CA, USA; ^7^ Boston University School of Public Health, Boston, MA, USA

## Abstract

**Background:**

Experiencing discrimination is associated with adverse physical and mental health outcomes that may contribute to the disproportionate rate of Alzheimer's disease and related dementias among Black women. Cognitive aging research rarely examines intersectional discrimination while capturing heterogeneity within groups. This study identified latent classes of intersectional discrimination among Black women to examine the association between everyday discrimination and episodic memory trajectories.

**Method:**

Participants were Black women (*M* age = 67.55, *SD* = 10.3) from the Health and Retirement Study (*N* = 1,074). Discrimination was assessed at baseline (2006/2008) using the Everyday Discrimination Scale (EDS) which asked participants to attribute their experience of discrimination to: ancestry, sex/gender, race, age, weight, disability, physical appearance, sexual orientation, and/or other. Latent class analysis of EDS attributions was used to identify classes of intersectional discrimination. Class membership was assigned by highest posterior probability. Episodic memory was assessed using a z‐score, standardized to baseline, calculated for five biennial waves (2008‐2016). Multiple‐group latent growth curve models examined associations between everyday discrimination and episodic memory trajectories, adjusting for age and years of education.

**Result:**

A four‐class solution categorized participants into classes of intersectional discrimination based on: (1) most attributes (1.77%); (2) ancestry, race, gender, and age (23.0%); (3) age, weight, and disability (6.13%); and (4) no or few attributes (69.1%). Greater everyday discrimination was associated with lower baseline memory in Group 4 (β = ‐.101; 95% CI: ‐.177, ‐.024), and no association was observed for other groups. Episodic memory declined over time across all groups, but the rate of change did not significantly differ by group (β range: ‐0.117 Group 1 to ‐.068 Group 2).

**Conclusion:**

This research attempted to account for the discrimination experienced by a heterogenous group of Black women. Baseline cognition was most strongly associated with discrimination among Black women reporting either the least frequent discrimination or fewest reasons for experiencing discrimination. For most of the sample, greater everyday discrimination was associated with lower episodic memory at baseline but not over time. There were no meaningful differences in memory decline over time across classes. Examination of associations with other aspects of cognition and risk of dementia is warranted.